# Electronic Coupling of Highly Ordered Perovskite Nanocrystals in Supercrystals

**DOI:** 10.1021/acsaem.1c03276

**Published:** 2022-02-22

**Authors:** Yingying Tang, Deepika Poonia, Marco van der Laan, Dolf Timmerman, Sachin Kinge, Laurens D. A. Siebbeles, Peter Schall

**Affiliations:** † Institute of Physics, University of Amsterdam, 1098 XH Amsterdam, The Netherlands; ‡ Optoelectronic Materials Section, Department of Chemical Engineering, Delft University of Technology, Van der Maasweg 9, 2629 HZ Delft, The Netherlands; § Graduate School of Engineering, Osaka University, Suita, Osaka 565-0871, Japan; ∥ Materials Research & Development, Toyota Motor Europe, B1930 Zaventem, Belgium

**Keywords:** perovskite, nanocrystals, assembly, supercrystals, carrier dynamics, coupling

## Abstract

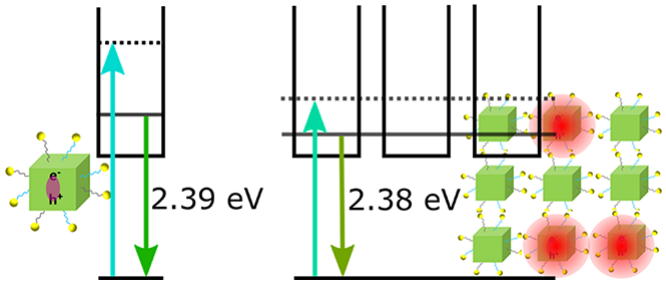

Assembled perovskite nanocrystals (NCs), known as supercrystals (SCs), can have many exotic optical and electronic properties different from the individual NCs due to energy transfer and electronic coupling in the dense superstructures. We investigate the optical properties and ultrafast carrier dynamics of highly ordered SCs and the dispersed NCs by absorption, photoluminescence (PL), and femtosecond transient absorption (TA) spectroscopy to determine the influence of the assembly on the excitonic properties. Next to a red shift of absorption and PL peak with respect to the individual NCs, we identify signatures of the collective band-like states in the SCs. A smaller Stokes shift, decreased biexciton binding energy, and increased carrier cooling rates support the formation of delocalized states as a result of the coupling between the individual NC states. These results open perspectives for assembled perovskite NCs for application in optoelectronic devices, with design opportunities exceeding the level of NCs and bulk materials.

## Introduction

Perovskite nanocrystals (NCs) display attractive structural and optical properties and have perspectives in a wide range of optoelectronic applications such as solar cells, lasers, light-emitting devices (LEDs), and photodetectors.^1−5^ Recent works have shown the controlled assembly of highly ordered NCs into so-called supercrystals (SCs), exhibiting novel optoelectronic properties distinct from their bulk material, opening new opportunities for applications of these structures.^6^ For example, a high photoluminescence (PL) efficiency for the SCs was maintained when compared to bulk perovskite materials, showing its potential for the fabrication of efficiently emitting layers for LEDs based on rigid or flexible substrates.^7^ The emergent electronic and excitonic states of the assembled SCs are central to these applications, as they could lead to bathochromic shift, miniband formation,^8−11^ or collective behavior.^12^ Upon assembly, the inter-NC coupling increases, causing the optical properties of the SCs to change as compared to the individual NCs. The formation of minibands in the superlattice is usually observed due to the electronic coupling between the NCs,^9^ which is concomitant with the lowering of the band gap energy, leading to red shift in the light emission; The latter can also arise from energy transfer between the packed NCs, and distinguishing these processes remains challenging.^13^ For SCs of perovskite NCs, an accelerated radiative decay, extension of the first-order coherence time, photon bunching, and delayed emission pulses with Burnham–Chiao ringing behavior at high excitation densities have been observed,^12^ indicating the occurrence of superfluorescence. A stronger coupling for packed NCs has later been supported by theoretical modeling.^14^ Furthermore, template-induced self-assembled CsPbBr_3_ NCs into two-dimensional (2D) SCs showed amplified spontaneous emission (ASE) under lower optical excitation fluences in the near-IR.^15^ These findings provide opportunities for many near-field and far-field applications for such coupled systems, such as nanoantennas and LEDs.

Besides the above exotic optical properties of SCs, the enhanced NC coupling in well-assembled SCs should ultimately lead to improved electronic properties crucial to device applications. The delocalization of excitons and miniband formation were inferred to explain the difference in decay time of the luminescence of CsPbBr_3_ SCs with cubic morphology, supercubes, whereas analysis of the ultrafast carrier dynamics did not show any considerable differences.^16^ More detailed studies of the optical properties and relation to ultrafast carrier dynamics are required to get a better understanding of the transition from NCs to SCs.

Here, we investigate the ultrafast carrier dynamics of SCs of perovskite NCs by femtosecond transient optical absorption (TA), and steady-state PL and absorption measurements, to characterize the excited states of the assembled NCs and their time-dependent occupation after photoexcitation. We focus on spherical SCs, which we term superballs (SBs),^17^ and employ TA measurements in the low-excitation regime, where the average number of absorbed photons per nanocrystal, ⟨*N*⟩, is below 0.10 to limit multiple exciton processes. We find a lower band gap energy and faster carrier cooling than that of the dispersed NCs, which we attribute to the increased density of states in the ordered NC assemblies, i.e., the SBs. This is further supported by the early TA dynamics, demonstrating a smaller biexciton binding energy in the assembled SBs, and the absence of a phonon bottleneck, both indicating the formation of bulk-like states in the SBs. These results are important for device applications, where device performance typically leans on the availability of band-like states for electronic transport and high carrier mobility.

## Materials and Methods

### Chemicals and Materials

All of the chemical reagents were at least of analytical grade and used without further purification. Lead(II) bromide (PbBr_2_, 99%), cesium carbonate (Cs_2_CO_3_, 99%), 1-octadecene (ODE, technical grade 90%), oleic acid (OA, technical grade 90%), and oleylamine (OAm, 90%) were all purchased from Sigma-Aldrich. 008-FS surfactant in FC-40 (5 wt %), and FC-40 were all purchased from RAN Biotechnologies. Toluene was obtained from Honeywell.

### Preparation of CsPbBr_3_ NCs

CsPbBr_3_ NCs were synthesized according to the widely used method developed by Protesescu.^18^ Cesium oleate was prepared by loading 0.814 g of Cs_2_CO_3_ into a 100 mL flask with 40 mL of ODE and 2.5 mL of OA, drying it for 1 h at 120 °C, and subsequently heating it under N_2_ to 150 °C until all Cs_2_CO_3_ had reacted with the OA. A 30 mL aliquot of ODE and 1.88 mmol of PbBr_2_ are dried under N_2_ and 120 °C. Dried 5 mL OAm and 5 mL OA are injected into the ODE solution, and the temperature is raised to 160 °C until all of the PbBr_2_ is dissolved. The earlier prepared cesium oleate is then heated to 100 °C, and 4 mL is injected in the 160 °C hot PbBr_2_ solution which is cooled in an ice–water bath 5 s later. The mixture was subjected to several purification cycles by centrifugation and redispersion in a solvent (i.e., toluene). The process was repeated 3 times.

### Self-Assembly of CsPbBr_3_ NCs

(1) A 12 or 60 μL aliquot of 008-FS in FC-40 (5 wt %) was injected into 588 or 540 μL of neat FC-40, forming 0.1 or 2 wt % 008-FS in FC-40, respectively.^17^
(2) CsPbBr_3_ NCs in toluene (5 mg/mL) was added into the above solution, followed by vortex mixing for 5 min.
(3) The above emulsion was kept stirring and evaporating at 20 °C for 3 days.
(4) After the emulsion was dried, the suspension was centrifuged to collect the SCs assemblies. The resulting perovskite NC SBs had a spherical morphology and showed a high degree of ordering.

### Optical Measurements

Scanning electron microscopy (SEM) imaging was conducted on FEI Verios 460 from FEI Co. using Cu grids as support materials. For SEM imaging, the sample was dropped cast onto the Cu grids and then dried under vacuum for a whole day.

To determine the difference in optical properties of ensembles of NCs and SBs, we performed absorption and PL measurements on the NC and SB dispersions in cuvettes with a light path of 1 cm as well as on the quartz substrate. Absorption/optical density spectra were determined using a LAMBDA 950 UV/vis/NIR spectrophotometer (PerkinElmer) by a transmission mode. The absorption spectra of the solvent (toluene and FC40) in cuvettes were measured separately and subtracted from the spectra to correct for solvent effects. PL measurements were performed with a Jobin Yvon FluoroLog spectrometer (Horiba). All spectra were corrected for the spectral sensitivity of the spectrometer.

### TA Measurements

TA measurements have been performed for both NCs and SBs on the colloidal dispersions in cuvettes with a pump excitation wavelength of 400 nm for four different pump fluences (1, 2, 3, and 5 μJ cm^–2^). The light path of cuvette is 1 cm. Light from an Yb:KGW oscillator (light conversion, Pharos SP), which produces 1028 nm, 180 fs pulses at a frequency of 5 kHz, is sent through an optical parametric amplifier (OPA) and a second harmonic module (light conversion, Orpheus) to generate the pump beam. Part of the light from the oscillator was sent through a sapphire crystal to generate a broadband spectra which was used as probe beam. The pump beam was guided through a mechanical chopper operating at 2.5 kHz and transmits every other pulse. The two beams overlap at the sample under a small angle (8°). The time delay *t*_pp_ between the two pulses was controlled by an automatic delay stage, and the intensity of the probe beam was determined by a detector (CMOS, Ultrafast Systems, Helios). The differential data (Δ*A*) was obtained by measuring the probe beam intensity with (*I*_on_) and without the pump pulse (*I*_off_), according to Δ*A* = −log(*I*_on_/*I*_off_). The TA data have been corrected for chirp.

## Results and Discussion

**Figure 1:**
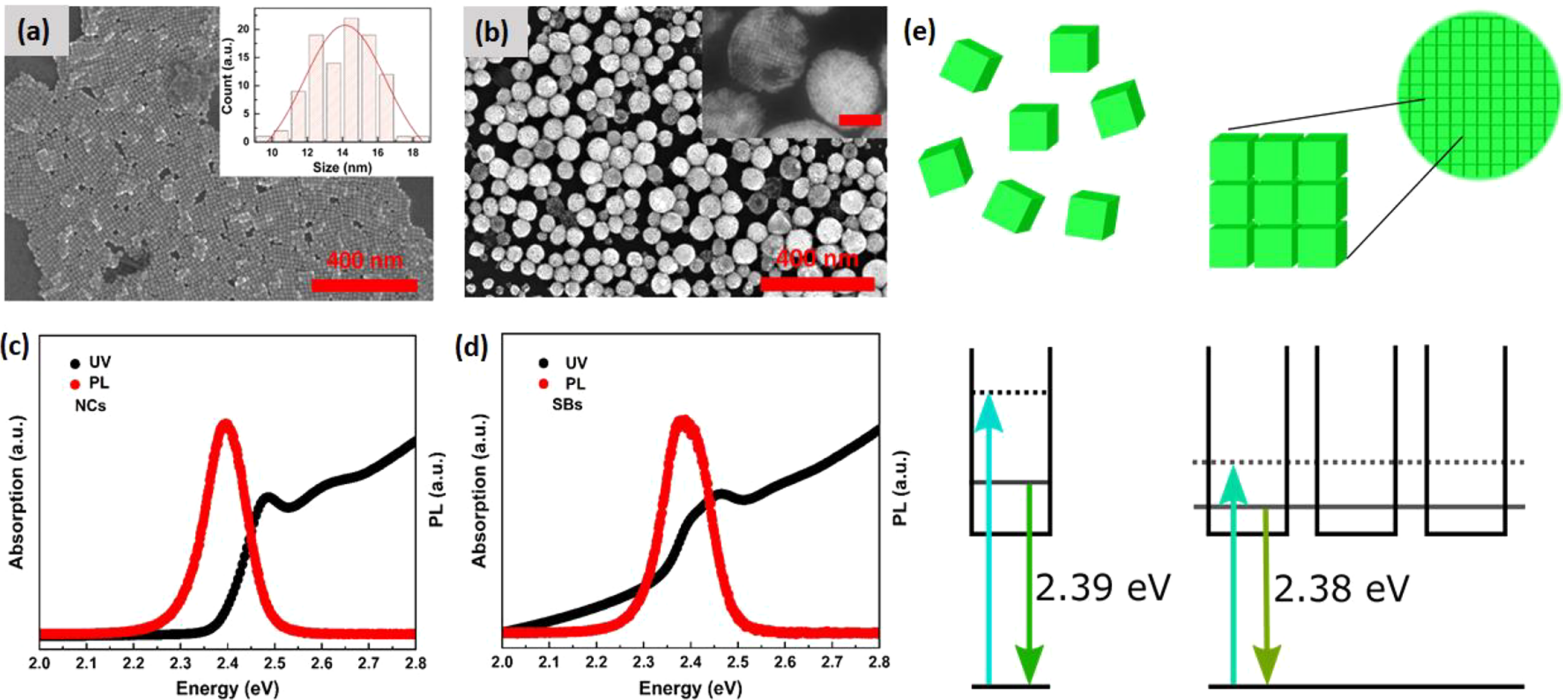
Properties of the NCs and SBs. (a, b) SEM images of NCs (a) and SBs (b). The inset in panel a shows the size distribution of the NCs, while the inset in panel b shows a higher magnification of the ordered NCs inside the SBs. Scale bars, 100 nm. (c, d) Absorption (black) and PL spectra (red) of colloidal NCs (c) and SBs (d). The fwhm of the PL for NCs and SBs are 104 and 116 meV, respectively. (e) Schematic of the absorption and emission states for NCs and SBs.

SEM images of CsPbBr_3_ NCs synthesized by the hot-injection method and SBs obtained from oil-in-oil emulsion assembly are shown in Figure 1a,b. The average size of the NCs is around 14 nm (Figure 1a), while the assembled SBs have an average size of around 90 nm (Figure 1b; for the size distribution, see Supporting Information (SI) Figure S1). The NCs are well-ordered within the SBs, as can be seen in the inset of Figure 1b. From the FFT images of the selected area, as shown in Figure S2, crystalline ordering can be identified from the appearance of four spots, indicating domains of cubic ordering in the SBs. The PL and absorption spectra of both NCs and SBs are shown in Figure 1c,d, respectively; the extracted peak values are listed in Table S1. A red shift in both absorption and PL spectra can be observed upon assembly of the NCs into SBs. Due to the stronger scattering of the relatively large SBs, there appears to be a sub-band-gap absorption signal. In Figure S3, we show the absorption spectra corrected for scattering assuming a λ^–4^ dependence. Keeping this in mind, we still observe a shoulder at about ∼60 meV below the main absorption peak. The main peak likely originates from individual, uncoupled NCs remaining in the suspension, whereas the newly introduced absorption feature around 2.4 eV originates from new energy levels due to the SB formation. The absorption transition energy was determined by locating the minimum of the second derivative of the absorption spectra (see Figure S3), and confirms the absorption feature at ∼60 meV below that of the NCs. The presence of two absorption edges in Figure 1d and Figure S3 then allows us to clearly delineate the red shift of the assembly with respect to the individual NCs. Furthermore, the PL peak red shifts by ∼10 meV and exhibits a slight line width broadening, which can be best seen in the overlaid PL spectra in Figure S4a. The PL peak of the SBs is approximately 10 meV below that of the NCs. We do note that the energies of the absorption and PL peaks of the SBs are close to values typically found for CsPbBr_3_ thin films, which is expected to be around 2.35 eV.^19,20^ Furthermore, while the Stokes shift for NCs is ∼80 meV, for the SBs it decreases to ∼20 meV, which is in line with the value of bulk CsPbBr_3_.^21^ A schematic of the differences in absorption and emission is given in Figure 1e. Baranov et al. observed a large red shift of an aged superlattice sample comprising 8 nm sized NCs as compared to the individual NCs, which they suggested to be related to bulk-like CsPbBr_3_ particles formed by fused NCs in the superlattice.^20^ The small red shift that we observe in the freshly produced SBs in solution suggests there is no such coalescence or bulk formation due to such an aging effect. This is further supported by the larger photon energy, although moderately, than that reported for bulk perovskites.^19,20^ Also high-resolution SEM images (Figure S2) show that the NCs in the SBs are clearly separated, displaying crystalline ordering and no coalescence of NCs. We have performed absorption and PL measurements for different dilutions (up to 100 times) of the SB suspensions in order to exclude any reabsorption effect (Figure S4b,c), as this could also give an apparent red shift.^22^ After drop-casting on the quartz substrate, the absorption and PL spectra of the SBs film are also similar to the colloidal solution (Figure S4d). Finally, we point out that the red shift is likely not due to superfluorescence, as it is not expected to survive thermal disorder at room temperature.^14^ We thus conclude that electronic coupling of the NCs is the most likely explanation for the red shift in PL and absorption in the SBs.

**Figure 2:**
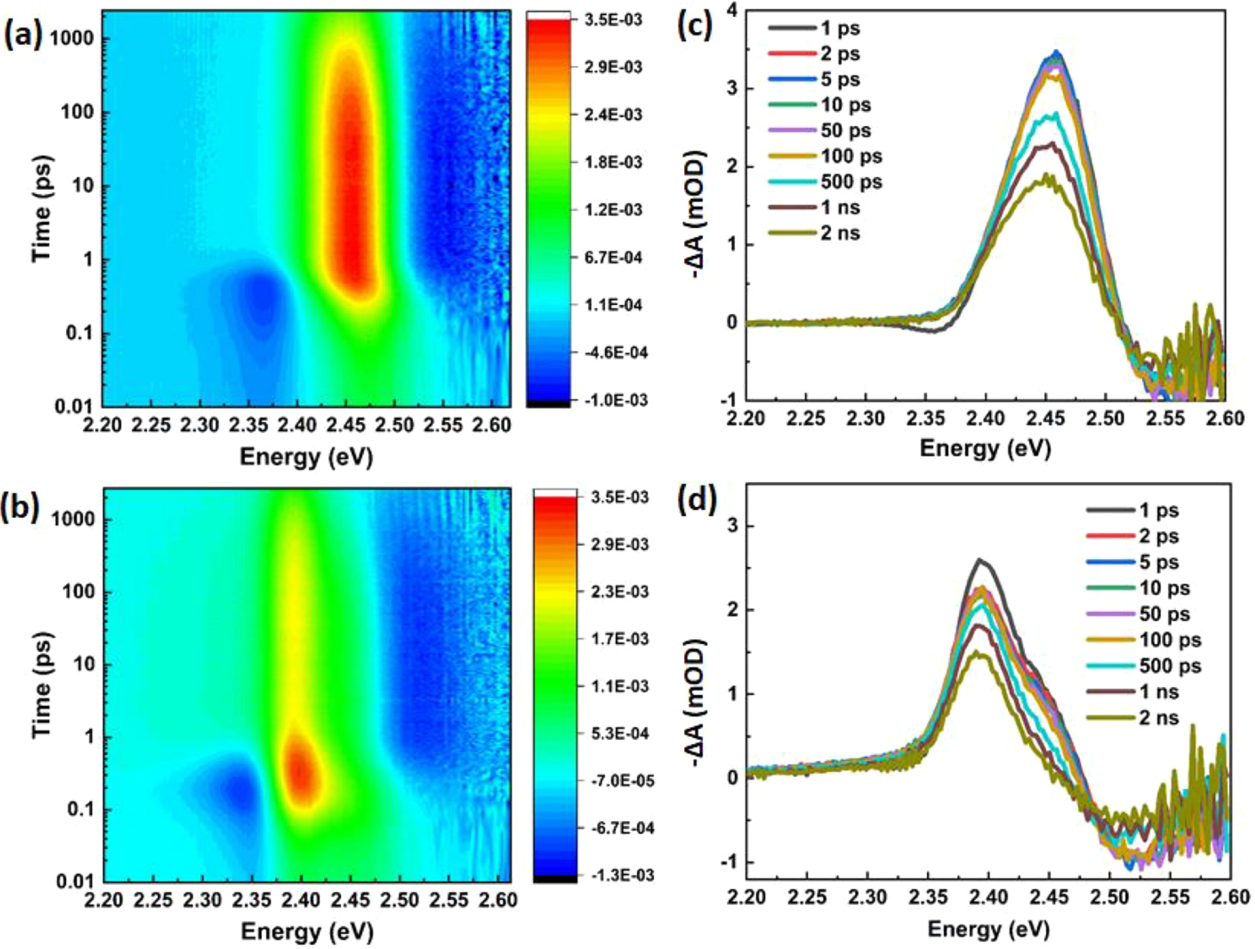
TA spectra of CsPbBr_3_NCs and SBs. (a, b) Contour plots of the differential absorption spectra for a pump wavelength of 400 nm for NCs (a) and SBs (b) for ⟨*N*⟩ = 0.10 (where, for example, 3.5E-03 represents 3.5 × 10^–3^). (c, d) Differential spectra for NCs (c) and SBs (d) for ⟨*N*⟩ = 0.10 at different delay times.

TA measurements were performed to determine the effects of NC assembly on the ultrafast carrier dynamics over a wide range of low pump photon fluences. Two-dimensional color maps show the differential absorption spectra for dispersed NCs and SBs, as a function of delay time in Figure 2a,b. They are obtained after an excitation with 3.1 eV photons (400 nm), and an excitation power of 5 μJ/cm^2^, corresponding to an average number of absorbed photons per NC, ⟨*N*⟩ = 0.10. Positive values—Δ*A* > 0 (red)—indicate ground state bleach (GB), while negative values—Δ*A* < 0 (blue)—indicate induced absorption (IA). The bleach peaks at 2.45 eV for the dispersed NCs, and 2.39 eV for the assembled SBs reflect reduction of the ground state occupation, which is observed to occur within 1 ps. In addition, at sub-band-gap energies an induced absorption feature can be observed for short times, which is typically explained by a biexciton effect,^23^ and which is sometimes absent in thin films.^24^ Above the band gap, the TA feature for energies larger than 2.5 eV is typically assigned to changes in the refractive index induced by the excited carriers,^25,26^ leading to reduction of the detected signal due to refraction or reflection. Comparing the TA maps for NCs and SBs, we clearly observe the red shift of the GB and IA features, as well as their different rise and decay times, which will be further discussed below. The red shift is most clearly observed in Figure 2c,d, where the TA spectra for different delay times are plotted.

### Early Time Carrier Dynamics

**Figure 3:**
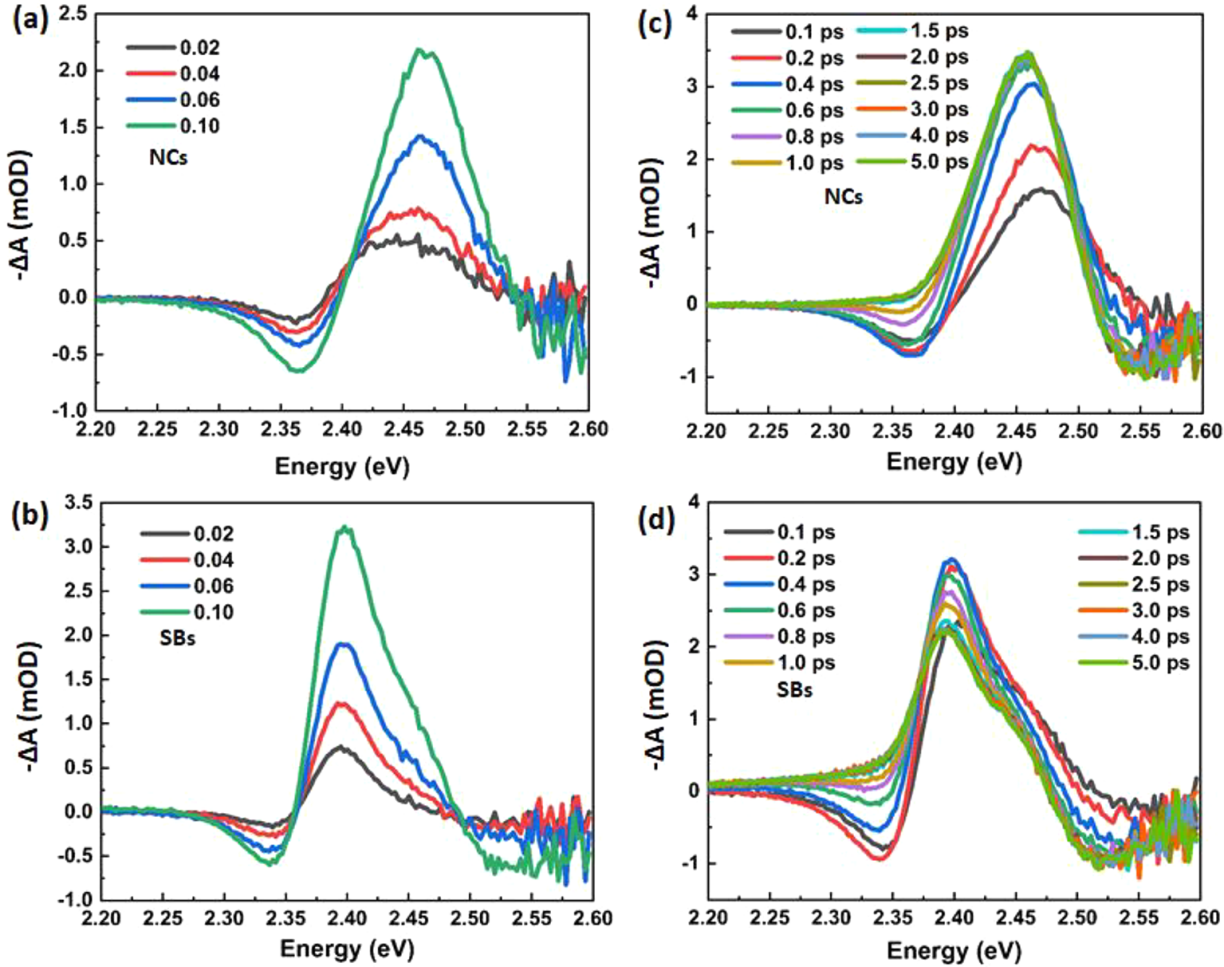
Transient optical spectra of CsPbBr_3_NCs and SBs at early times. (a, b) Differential spectra for NCs (a) and SBs (b) at 0.2 ps time delay after optical excitation for ⟨*N*⟩ = 0.02, 0.04, 0.06, and 0.10. (c, d) Differential spectra for NCs (c) and SBs (d) at ⟨*N*⟩ = 0.10 at different delay times.

Pump fluence-dependent TA spectra of both NCs and SBs for *t*_pp_ = 0.2 ps are depicted in Figure 3a,b. The GB amplitude is proportional to the number of photogenerated carriers, which increases with the pump photon fluence. While the NCs show a clear blue shift with increasing values of ⟨*N*⟩ in Figure 3a, indicating an increasing effective carrier temperature of the quasi-thermalized carrier distribution,^27,28^ this effect is not obvious for the SBs (Figure 3b). Furthermore, the energy difference between the sub-band-gap absorption feature and the bleach peak is considerably larger for NCs than for SBs. This energy difference is commonly assigned to band gap renormalization or biexciton effects and will be analyzed in more detail further in this work.

**Figure 4:**
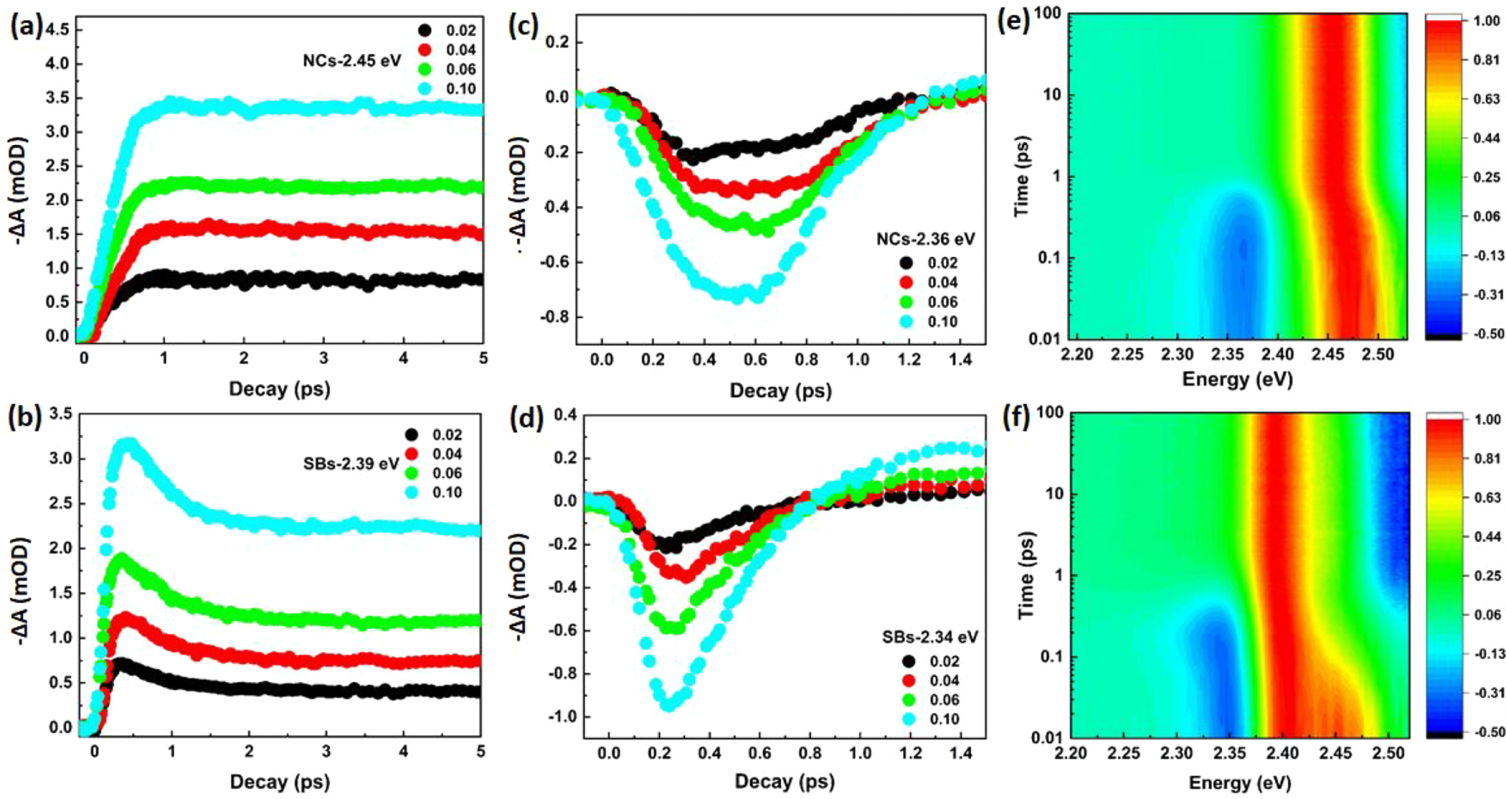
(a, b) Temporal traces showing the early TA kinetics at 2.45 eV for NCs and at 2.39 eV for SBs, respectively, at ⟨*N*⟩ = 0.02, 0.04, 0.06, and 0.10 (see legend). (c, d) IA peaks for NCs at 2.36 eV and SBs at 2.34 eV at early time scale for the same fluences. (e, f) Contour plot of the normalized differential absorption spectra for NCs and SBs, respectively, at ⟨*N*⟩ = 0.10.

In the initial TA spectra taken at different pump–probe delay times 0.1 < tpp < 1 ps (Figure 3c,d), a number of interesting phenomena can be observed. (1) Both NCs and SBs show a typical red shift at early time scales, which results from non-equilibrium carriers cooling toward the band gap and in this way reducing their effective temperature, although for SBs this is only a minor effect. (2) The sub-band-gap photon-induced absorption (PIA) feature, which is initially high, decreases on the same time scale and is therefore attributed to the presence of hot carriers. After relaxation of the carriers a strong bleach signal around the band edge appears and the PIA feature disappears, as a direct result of the band-edge state filling.^23^ (3) For NCs, the increase of the GB signal and the decrease of sub-band-gap PIA signal are monotonic during the first 1 ps (Figure 4a). Contrarily, the GB signal of the SBs increases up to about 300 fs and then starts to decrease quickly, while the sub-band-gap PIA feature decreases considerably faster than that observed for the NCs. From the initial time traces taken at the peak of the GB in Figure 4b, it can be seen that the rise time for the SBs is considerably faster than that for the NCs. Because this rise time is associated with the cooling of carriers to the band edge, it implies that carrier cooling is much faster in the SBs. This is supported by the sub-band-edge PIA time traces (at 2.36 and 2.34 eV for NCs and SBs, respectively) as shown in Figure 4c,d, which decrease considerably faster due to the faster filling of the band-edge states.^26^ From fits of the rise of the GB signal we find a carrier cooling time (during which the GB signal rises to (1 – *e*^–1^) of the plateau) of ∼200 fs for the NCs, while for the SBs it is too fast to determine accurately, as the signal increases on the same time scale as the laser pulse width (Figure S5). The same effect can be seen in the normalized contour plot of the TA signal in Figure 4e,f. The energy of the peak of the bleach increases with carrier temperature, which decreases much faster to lower energy in the SBs. We note that a small part of the TA signal in the SBs originates from individual, uncoupled NCs. This is visible in the shape of the TA spectra at long delay times, where the high energy shoulder originates from the individual NCs (see Figure S6). Due to this contribution, it is not possible to determine accurate values of the carrier temperature. Furthermore, it will give a contribution to Figure 4f on the high-energy side, so that carriers seemingly cool slower. However, even with this contribution it is obvious that the effective carrier temperature decreases on a much faster scale. This supports the idea that the SBs consist of effectively coupled NCs and behave more bulk-like. The bulk character of the SBs reduces the phonon bottleneck, which occurs in lead-halide perovskite quantum dots.^29^ Finally, we note that the GB of the SBs has an initial fast decaying component with a decay time of 650 ± 30 fs. This component is independent of the pump fluence and thus not likely related to carrier–carrier interactions. We consider this component to be due to trap states that are inflicted by the SB formation process and which remove about 30% of the excitons within a few picoseconds. This process likely is also largely responsible for the decrease of the photoluminescence quantum yield from 50.8 to 28.2% upon the formation of SBs that was observed in our previous work.^17^

### Biexciton Properties

The initial below-band-gap PIA feature can be assigned to an energy shift due to a biexciton effect.^23,30,31^ This is due to the Coulomb interaction between the exciton generated by the pump pulse and the exciton that is generated by the probe pulse. This interaction shifts the lowest optical transition compared to the transition energy in the absence of the first exciton, *E*_g_, by the biexciton energy Δ_*XX*_. Values of the biexciton binding energy have been determined for similarly sized NCs in the moderate confinement regime to be 25–40 meV by two-dimensional electronic spectroscopy,^32^ ∼35 meV by means of TA measurements,^23,33^ and ∼40 meV from the biexciton-related emission.^31^ These values are considerably larger than the biexciton energy expected for bulk CsPbBr_3_, which is below 10 meV.^34^

**Figure 5:**
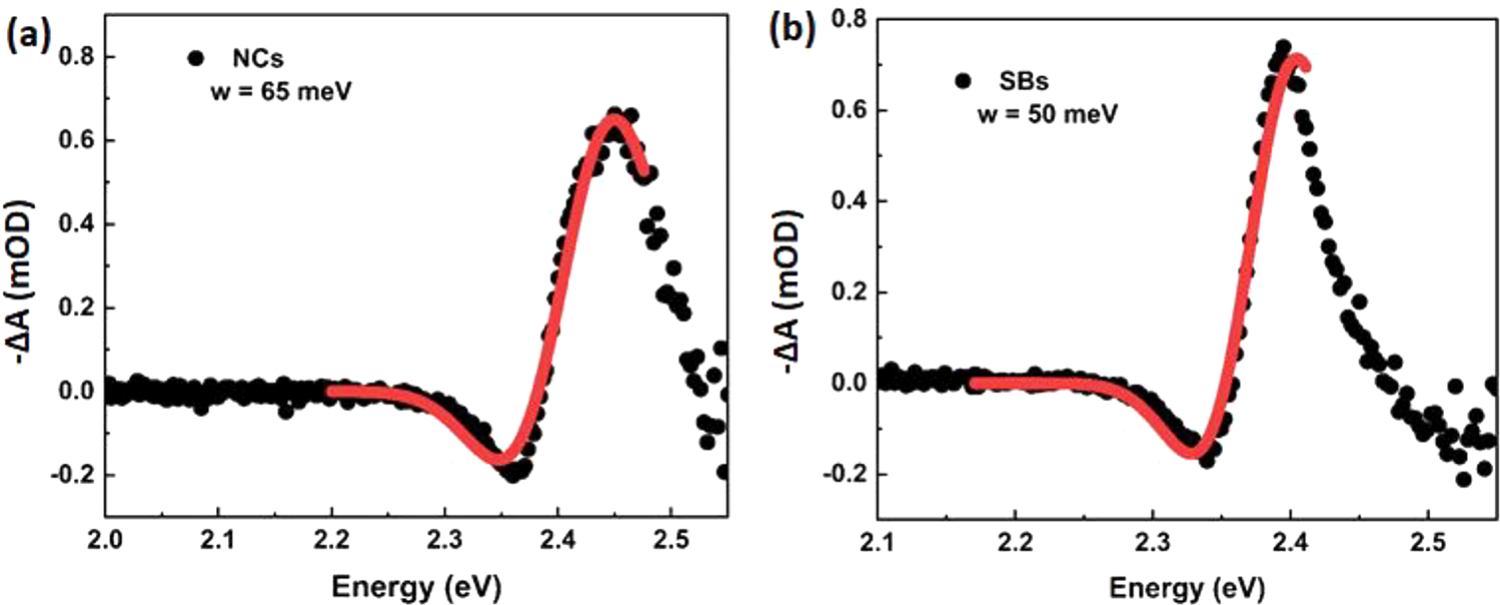
(a, b) TA spectra at delay time of 0.2 ps, where the solid lines are obtained by fitting a superposition of two Gaussians. See the SI for details.

We have estimated the biexciton energy Δ_*XX*_ from the differential absorption spectra obtained after photoexcitation, at delay time of *t*_pp_ = 0.2 ps, where the PIA peak has its maximum. The data of the differential absorption spectra are depicted in Figure 5a,b for NCs and SBs, respectively. These data are described by a fit of the sum of two Gaussian functions representing the induced absorption *E*_g_ + Δ_*XX*_ and the absorption bleach at *E*_g_,^23,35^
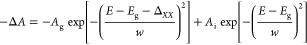
1where *A*_i_ and *A*_g_ are the amplitudes of the induced absorption and the ground state bleach, *E*_g_ the band gap as determined from the GB at long delays, and *w* the width of the first exciton resonance as obtained from the linear absorption spectra (see the Supporting Information). From this fitting procedure we find values of the biexciton energy of Δ_*XX*,NC_ = ∼−50 meV for the NCs and Δ_*XX*,SB_ = ∼−20 meV for the SBs. The slightly larger value for the NCs than what is found in literature could be a result of the excess photon energy used for excitation. For CsPbI_3_ NCs it has been shown that the interaction is dependent on the excess energy of the initially created hot exciton and reaches a constant level above the threshold excess energy, *E*_Ex_ ∼ 0.3 eV above the band edge, where Δ_*XX*_ is 1.6 times larger than that for *E*_Ex_ = 0.05 eV.^35^ While such a hot biexciton effect has not been explicitly quantified for CsPbBr_3_ quantum dots, it is conceivable to occur at the excess energy of *E*_Ex_ = 0.7 eV that is employed here, confirming the formation of hot biexcitons as concluded from their rapid disappearance on the carrier cooling time scale. Importantly, the lower biexciton binding energy of the SBs compared to that of the NCs further supports a lower degree of confinement due to more delocalized excitons.

### Longer Time Dynamics

Finally, we have analyzed the kinetics of the single exciton bleach signal for longer pump–probe delay times, *t*_pp_, for both NCs and SBs for ⟨*N*⟩ = 0.10 (see Figure S7). The TA of the NCs is best fitted with a small fast component of 330 ± 40 ps and a longer decay of 5.3 ± 0.3 ns. The origin of the small fast component is not clear as it is about an order of magnitude longer than the biexciton Auger recombination time^23^ and also is not expected to occur for these low excitation fluences. The SBs show a clear single-exponential decay after the initial very fast (650 fs) component which is fitted with a decay constant of 4.5 ± 0.1 ns. The longer decay time values are in line with the PL lifetime as obtained from time-resolved PL dynamics shown in Figure S8, of 6.4 and 5.2 ns, respectively.

## Conclusion

Assembled perovskite NCs in the form of superballs, prepared through an oil-in-oil emulsion templating method, show signatures of the emergence of new bulk-like electronic states as determined by systematic TA and optical measurements. The latter show a red shift in both absorption and PL spectra for the assembled as compared to the dispersed NCs, indicating states with lower energies in the SBs. The same red shift is observed in the bleach signal of the TA measurements. The rise time of the bleach signal for the SBs is shorter than that of the NCs, indicating that hot carrier cooling is considerably faster in the SBs. This is corroborated by the decay kinetics of the absorption features and demonstrates a reduction of the phonon bottleneck due to an increased density of states. The biexciton, formed by the initially hot-pump-generated exciton and the probe-generated exciton, exhibits a significantly smaller binding energy for the SBs (∼20 meV) than for the NCs (∼50 meV), further supporting the formation of more bulk-like states in the former. We expect that the coupling of the NCs within the SBs can be further increased by replacing the long OA ligands by shorter and more conductive ligands. These ordered perovskite NC assemblies in the form of SBs hence feature interesting prospects for fabricating optoelectronic devices.

## Supporting Information Available

STEM images, optical measurements (UV, PL, PL QY, and lifetimes), and additional analysis and discussions (PDF)
