# Supplementary material for: Electronic Coupling of Highly Ordered Perovskite Nanocrystals in Supercrystals
Source: ACS Appl Energy Mater. 2022 Feb 22;5(5):5415–22. doi: 10.1021/acsaem.1c03276 (PMC9131308; doi:10.1021/acsaem.1c03276)
Supplement: Supplementary file 1 — ae1c03276_si_001.pdf [file ae1c03276_si_001.pdf]

Supplementary information for

“Electronic Coupling of Highly Ordered Perovskite Nanocrystals in Supercrystals”

Yingying Tang,[a] Deepika Poonia,[b] Marco van der Laan,[a] Dolf Timmerman,[c] Sachin Kinge,[d] Laurens D. A. Siebbeles,[b] Peter Schall [a*]

[a] Institute of Physics, University of Amsterdam,1098 XH Amsterdam, The Netherlands

[b] Optoelectronic Materials Section, Department of Chemical Engineering, Delft University of Technology, Van der Maasweg 9, 2629 HZ, Delft, The Netherlands

[c] Graduate School of Engineering, Osaka University, Suita, Osaka 565-0871, Japan

[d] Materials Research & Development, Toyota Motor Europe, B1930 Zaventem, Belgium

Calculation of average occupancy, <*N*>

The average number of absorbed photon per NC, the occupancy <N>= σ $j_{h\nu}$, with σ the absorption cross-section of pump laser photons and $j_{h\nu}$ the pump photon fluence (number of photons/cm^2 per pulse). The pump photon fluence $j_{h\nu}$ was obtained from the measured pump power for a laser repetition rate of 2.5 kHz and a pin hole diameter of 2 mm. The absorption cross-section was obtained from the literature, 1* 10^{-14} cm^2.[1]

Determination of excitonic levels

Figure S1 shows the absorption for both NCs and SBs. These absorption spectra have been corrected for scattering by subtracting a λ^{-4} dependent function fitted to the below bandgap part of the spectra. At the top of the figure the second derivative of the absorption spectra are displayed. The discrete exciton absorption levels in $CsPbBr_3$ NCs and SBs can clearly be observed in the second derivative spectrum by the negative peaks, and these are used to determine the transition energy and width. The lowest energy negative peaks at 2.47 eV and 2.40 eV are attributed to the first exciton resonance for NCs and SBs, respectively, whereas there is also a NC-related excitonic feature in the SBs at 2.46 eV. The width is given by the width of the negative peaks at α” = 0, and gives values of 65 and 50 meV for the first exciton resonances of NCs and SBs, respectively.

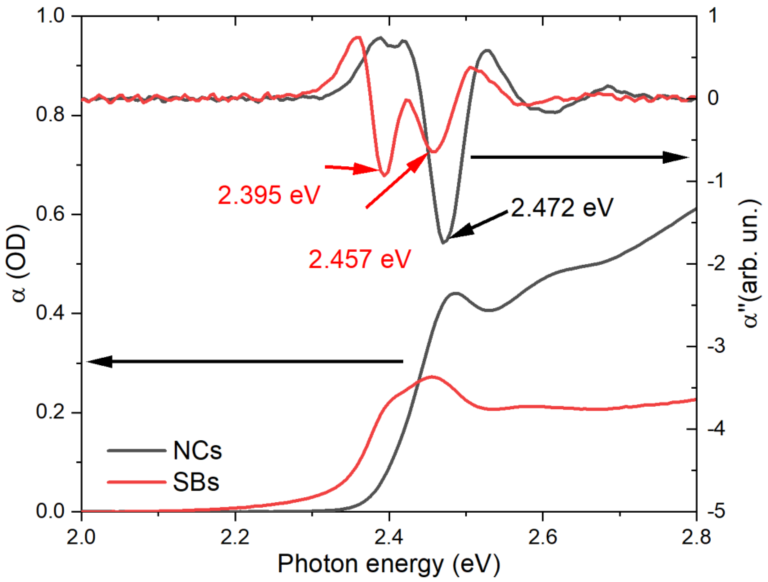

Figure S1. Linear absorption and second derivative for NCs (black curve) and SBs (red curve), respectively.

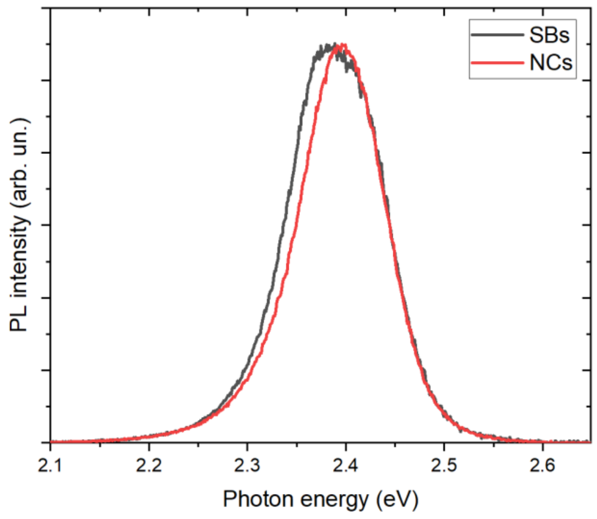

Figure S2. Normalized PL spectra for NCs and SBs showing redshift of ~10 meV for the SBs.

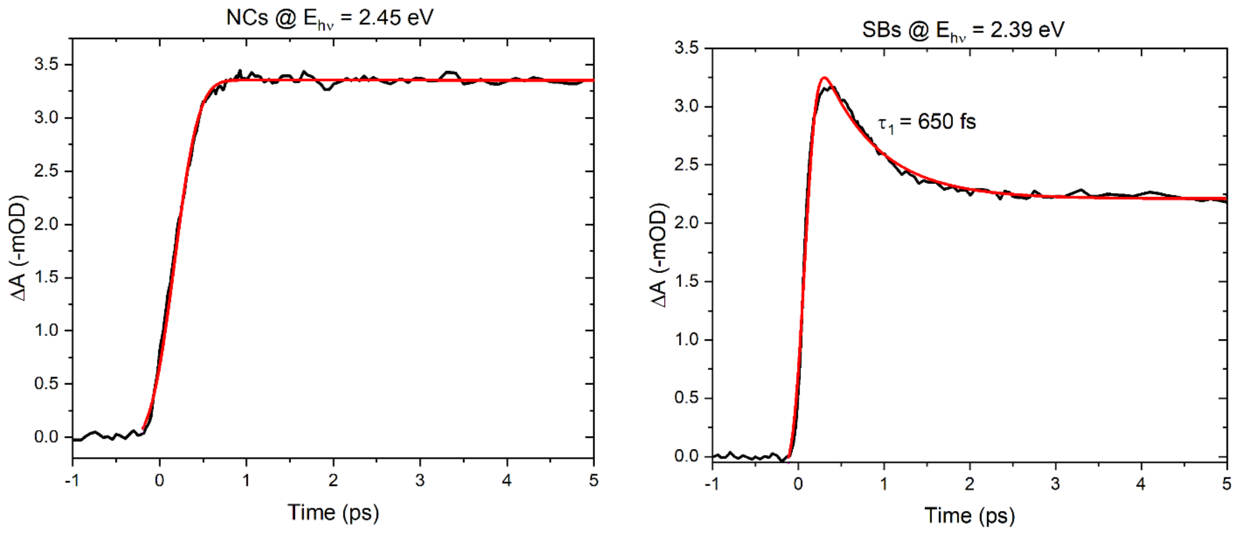

Figure S3. Fits of the intial rise and decay of the bleach signal for NCs and SBs for <*N*> = 0.10 with an exponential rise and double exponential decay function convoluted with the system response function.

Fitting and substraction of components of NCs from SBs

From the linear absorption spectrum of the SBs it was noticed that there is a contribution of (uncoupled) NCs as well. Similarily, the TA spectra show a signal related to NCs, as can be seen by the shoulder at larger energies (**Figure S4a**). The contribution hereof was determined by fitting two Gaussian functions at ~2.39 eV and ~2.44 eV for <*N*> of 0.02, 0.04, 0.06, 0.10 respectively. This contribution was subsequently subtracted from the spectra in order to resolve differences originating from the occupation <*N*> (**Figure S4b, S4c**).

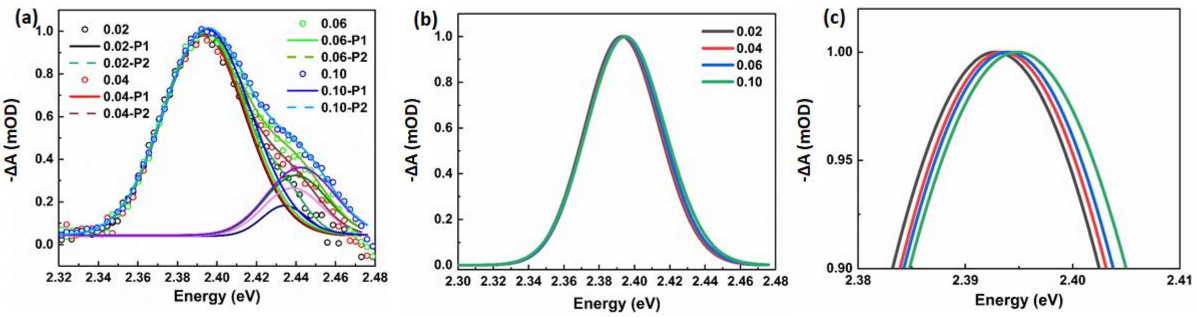

Figure S4. (a) Normalized differential spectra with Gaussian peaks fitting for SBs at 500 ps time delay with ⟨*N*⟩ = 0.02, 0.04, 0.06 and 0.10. **(b)** Normalized differential spectra for SBs at 500 ps time delay with ⟨*N*⟩ = 0.02, 0.04, 0.06 and 0.10 after substracting the signal of NCs (Peak 2: P2). **(c)** Zoom-in of Figure b.

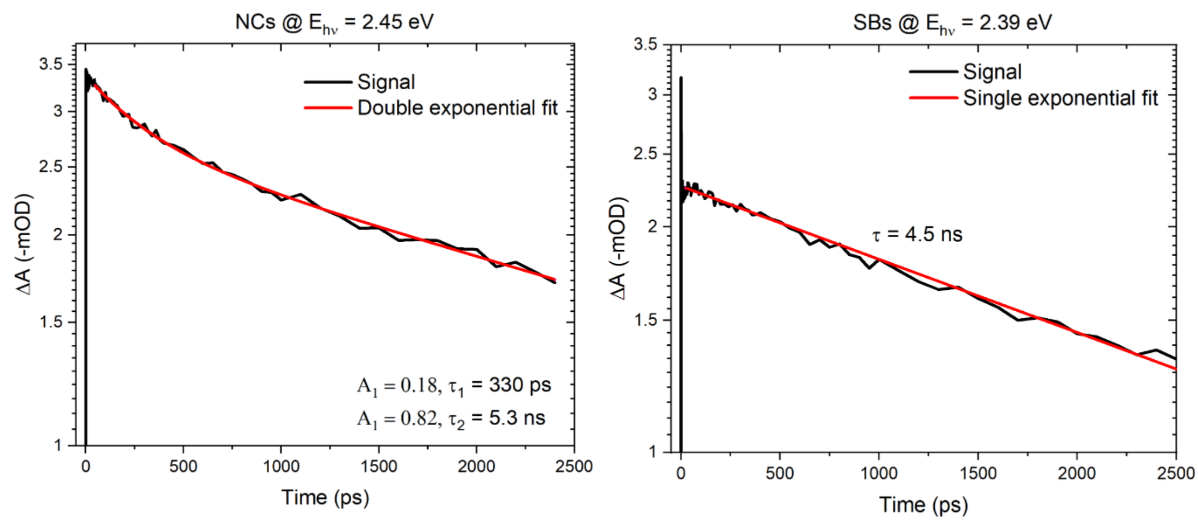

Figure S5. Bleaching dynamics for longer times for NCs with double exponential fit and SBs with single exponential fit.

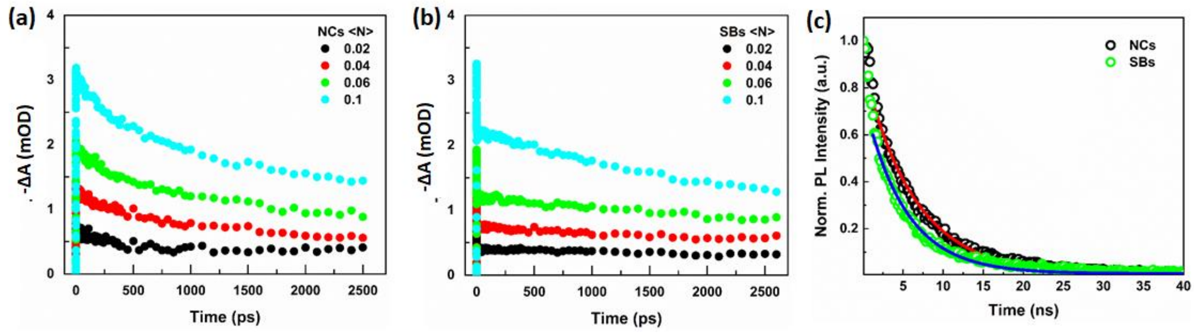

Figure S6. (a,b) Bleaching decay for NCs, 2.45 eV and SBs, 2.39 eV at different pump fluences. (c) Time-resolved PL decay for NCs and SBs.

Table S1. Energy peaks for NCs and SBs at delay time of 500 fs.

	**UV**	**PL**
**NCs**	2.47 eV	2.39 eV
**SBs**	2.45 eV, 2.40 eV	2.38 eV

References

1. Mondal, A.; Aneesh, J.; Ravi, V. K.; Sharma, R.; Mir, W. J.; Beard, M. C.; Nag, A.; Adarsh, K. V. Ultrafast Exciton Many-body Interactions and Hot-phonon Bottleneck in Colloidal Cesium Lead Halide Perovskite Nanocrystals. *Phys. Rev. B* **2018**, *98*, 115418.
